# Cucurbit[7]uril-based high-performance catalytic microreactors†
†Electronic supplementary information (ESI) available. See DOI: 10.1039/C8NR02900H


**DOI:** 10.1039/c8nr02900h

**Published:** 2018-07-27

**Authors:** Xiaohe Ren, Ziyi Yu, Yuchao Wu, Ji Liu, Chris Abell, Oren A. Scherman

**Affiliations:** a Melville Laboratory for Polymer Synthesis , Department of Chemistry , University of Cambridge , Lensfield Road , Cambridge , CB2 1EW , UK . Email: oas23@cam.ac.uk ; Fax: +44 (0)1223 334866; b Department of Chemistry , University of Cambridge , Lensfield Road , Cambridge , CB2 1EW , UK . Email: ca26@cam.ac.uk ; Fax: +44 (0)1223336455

## Abstract

Catalytic microreactors manufactured using microfluidic devices have received significant research interest in recent years. However, little attention has been paid to immobilising metallic nanoparticles (NPs) onto microchannel walls for high efficiency catalytic reactions. We demonstrate a facile preparation of cucurbit[7]uril-based catalytic microreactors, where metallic NPs are immobilised onto microchannels *via* supramolecular complexation with methyl viologen@cucurbit[7]uril (CB[7]). These microreactors exhibit a remarkable catalytic activity owing to the substantially high surface area to volume ratio of the microchannels and metallic NPs. Superior to most conventional heterogeneous catalytic reactions, separation post reaction and complicated recycling steps of the catalysts are not required. Moreover, CB[7] can complex a variety of metallic NPs to its portal, providing a multifunctional high-performance *in situ* catalytic platform.

## Abstract 2

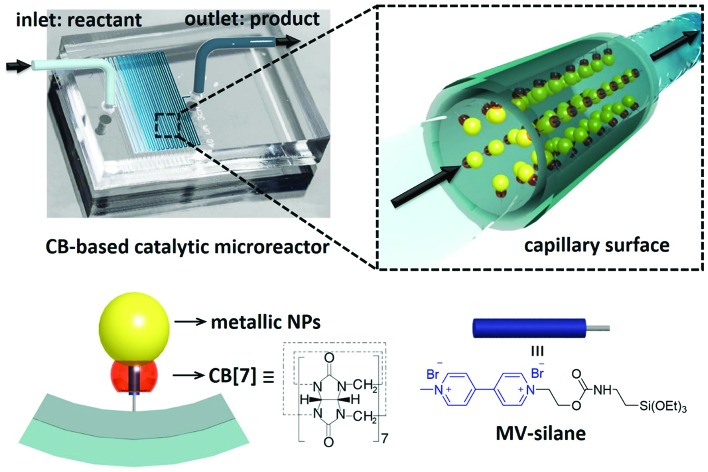
Fabrication of cucurbit[*n*]uril-based catalytic microreactors through the immobilisation of metallic nanoparticles onto microchannels.

## 

Microfluidics has received much attention over the past few decades in a broad range of fields,^1^–^4^ including chemistry,^5^,^6^ biology,^7^,^8^ pharmaceutics,^9^ physics^10^ and materials science.^11^ In particular, flow chemistry using microfluidic devices has attracted great interest, on account of a variety of distinct advantages.^9^,^12^,^13^ For instance, a significant surface area to volume ratio in a microreactor (10 000 to 50 000 m^–1^) can dramatically enhance the mass transport efficiency of reactants and products.^12^ In addition, a small channel diameter (10–1000 μm) results in largely improved heat transfer for isothermic catalytic reactions, allowing high selectivity towards desired products.^9^,^12^,^14^ Catalytic microreactors are one of the most popular topics in flow chemistry.^12^,^15^ Various approaches to fabricate catalytic microreactors have been reported,^15^–^21^ among which the most advantageous has been functionalisation of the microchannel surface using catalysts.^22^,^23^ This surface functionalisation approach could substantially minimise the mass transfer resistance, avoiding adverse pressure drops or blockages of the microchannels, problems commonly observed in other approaches.^9^,^12^ However, investigation of surface functionalised catalytic microreactors has been mainly focused until now on the immobilisation of organocatalysts^24^,^25^ and organometallic catalysts.^26^,^27^ Less effort has been devoted to immobilising metallic nanoparticles (NPs) onto microchannel surfaces.^9^,^27^,^28^ The state-of-the-art approaches to prepare such microreactors suffer from shortcomings of high temperature and harsh conditions,^9^,^12^,^27^,^29^ as well as inevitable leaching of the metallic NPs due to fragile attachment.^9^,^22^,^28^

Herein, we demonstrate a facile fabrication route to multifunctional catalytic microreactors based on CB[*n*] host–guest chemistry (illustrated in Fig. 1). CB[*n*]s are a family of symmetric barrel-shaped host molecules, which are capable of selectively encapsulating small guest molecules in their cavity (*K*_a_ up to 10^15^ M^–1^).^30^–^32^ Moreover, the portal of CB[*n*] exhibits strong binding affinity towards various metallic NPs (*e.g.* gold, palladium, platinum, copper and silver).^33^,^34^ By functionalising the microchannel with methyl viologen (MV)–silane@CB[7] complexes, a variety of metallic NPs could be readily loaded and immobilised onto the microchannel wall. These CB[7]-based catalytic microreactors exhibit higher catalytic activity than most single-site heterogeneous NP catalysts supported by mesoporous silica,^35^–^37^ carbon,^36^–^38^ polymer networks,^36^,^37^,^39^,^40^ and other substrates,^36^,^37^,^41^,^42^ while avoiding the inconvenience of post-reaction catalyst separation as well as leaching.^35^–^42^

**Fig. 1:**
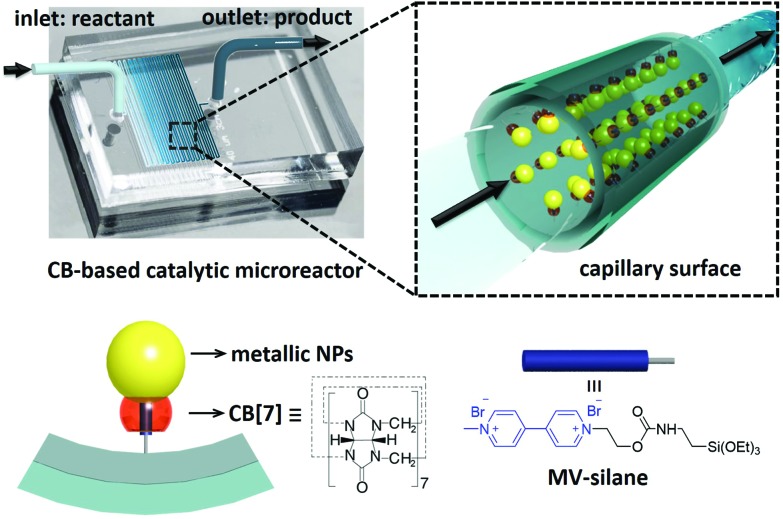
Schematic illustration of a cucurbit[7]uril-based high-performance catalytic microreactor.

The microfluidic devices were prepared through well-established soft lithography using polydimethylsiloxane (PDMS).^4^ The CB[7]-based catalytic microreactors were fabricated in two steps. First, after activation of the microchannel by oxygen plasma, an aqueous solution of an MV–silane@CB[7] inclusion complex was injected into the microchannel at a flow rate of 300 μL h^–1^ for 2 h. The methyl viologen groups form strong 1 : 1 inclusion complexes with CB[7], with an association constant *K*_a_ of 3.3 × 10^5^ M^–1^ (ESI Fig. S3†). Meanwhile, the silane moieties on MV–silane covalently attached and became immobilised onto the activated microchannels. The second step was the flow of the metallic NP solution into the microchannel at a flow rate of 200 μL h^–1^ for 1 h. The PDMS microchannel can be made with different geometries or lengths depending on the requirement for the particular catalytic reaction (ESI Fig. S2†). Au (6.8 ± 2.1 nm) and Pd NPs (3.7 ± 0.8 nm) were chosen as example metallic NPs to be immobilised inside the microchannels on account of their excellent catalytic activity.^43^,^44^

Fig. 2b and c show SEM images of the internal channel of the CB[7]-based Au NP catalytic microreactor. The loaded Au NPs can be clearly observed suggesting their immobilisation. AFM images of the Au NP microreactor are shown in Fig. 2d–f. According to the 3D view and topography images, Au NPs formed a monolayer inside the microreactor. The height of Au NPs in the AFM profile (Fig. 2f) is consistent with the size of the Au NPs obtained from TEM (6.8 ± 2.1 nm, ESI Fig. S4†). Moreover, it was observed that by increasing the concentration of injected Au NP solutions (*e.g.* from 1 wt% to 5 wt%), the density of the immobilised Au NPs increased (from 0.11 ± 0.06 to 0.20 ± 0.05 nm^–2^, ESI Fig. S12†). The immobilisation of Pd NPs in a CB[7]-based catalytic microreactor was also confirmed, with the immobilised Pd NP density of 0.15 ± 0.07 nm^–2^ deposited from a 1 wt% solution (ESI Fig. S6, S7 and S11†).

**Fig. 2:**
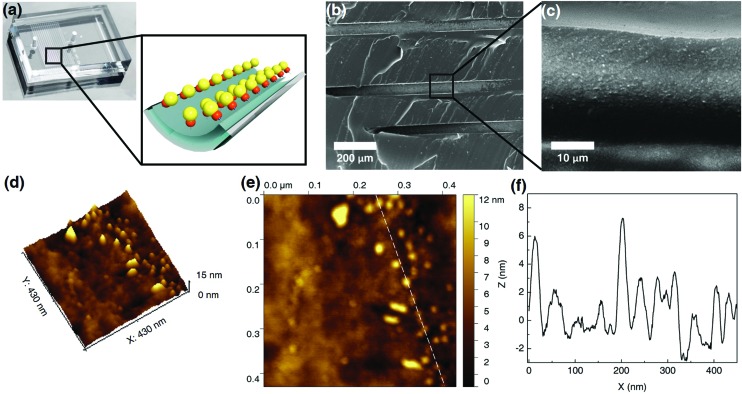
(a) Illustration of the internal structure of a microreactor. (b) and (c) SEM images of the internal surface of a CB[7]-based Au NP catalytic microreactor, where the immobilisation of Au NPs is clearly observed. (d) AFM 3D view of the Au NP microreactor internal surface. (e) AFM topography image of the Au NP microreactor internal surface. (f) Profile of the dashed white line in (e), where the height of Au NPs is consistent with the size of Au NPs obtained from TEM (6.8 ± 2.1 nm).

The catalytic activity of the Au NP microreactor with a density of 0.11 ± 0.06 nm^–2^ was examined with three reactions: reduction of nitrobenzene and 4-nitrophenol, as well as the catalytic oxidation of 3,3′,5,5′-tetramethylbenzidine (TMB), as illustrated in Fig. 3a–c and ESI Table S1.† To investigate the importance of the MV–silane@CB[7] complexes immobilised on the surface of the microreactor, control experiments were carried out using: (i) a blank PDMS microchannel, (ii) a control channel (prepared without injection of the CB[7] solution) and (iii) the reaction carried out using “bench” conditions.

**Fig. 3:**
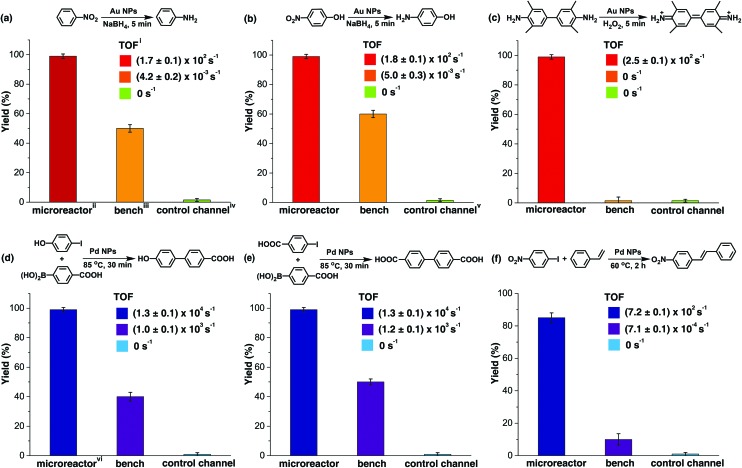
Summary of representative reactions catalysed by CB[7]-based Au/Pd NP catalytic microreactors. (a), (b) and (c) show the yield and turnover frequency (TOF) of three typical reactions catalysed by the Au NP microreactor, control channel and reactions on bench. (e), (f) and (g) show the results for Pd NP catalysed reactions, demonstrating the versatility of the microreactor. The microreactors led to high purity products with high yields (85% to 99%) in all reactions, while the bench reaction gave rise to comparably low yield products (10% to 60%) with various byproducts. The control channel showed almost no catalytic ability due to leaching of catalysed particles during the reactions. ^i^ The resultant turnover frequency suggests remarkable catalytic activity of the microreactors, remaining unchanged for more than 300 h at a flow rate of 200 μL h^–1^. The solvent system for reactions in (a) and (b) was water, 1 : 1 water/ethanol for (c), (d) and (e) and 1 : 2 water/DMF for (f). ^ii^ Au NP reactions in microchannels were carried out at a flow rate of 200 μL h^–1^. The yield was determined from UV-vis absorption analysis. ^iii^ Bench reactions were carried out under approximately similar conditions, using free metallic NPs as the catalyst instead. ^iv^ Control channels were prepared by injecting only MV–silane and metallic NP solutions into the microchannel, but without CB[7] solutions. The first run gave rise to 50% yield; however, this quickly decreased to 0% in the subsequent reaction cycles due to severe leaking of the particles. ^v^ The first run gave rise to 60% yield and then quickly decreased to 0% in the following cycles. ^vi^ Pd NP reactions in microchannels were carried out at a flow rate of 100 μL h^–1^. The yield was determined from HPLC analysis.

The catalytic reduction of nitrobenzene by NaBH_4_ in the presence of Au NPs is a typical test reaction yielding aniline, which is an important chemical widely used in the fine chemical, agrochemical and pharmaceutical industries.^45^ As shown in Fig. 3a and ESI Fig. S13,† the microreactor gave a 99% yield in 5 min, while 0% yield was observed for the reaction in the blank microchannel and only 50% for the reaction carried out on the bench under approximately similar conditions. Importantly, 50% yield was observed in the control channel on the first run; however, this quickly decreased to 0% in the subsequent reaction cycles. This initial moderate level of catalytic activity might be explained by the formation of Au agglomerates arising from electrostatic attraction between positively charged MV groups and the negatively charged Au NP surface (ESI Fig. S5c and S9†). Au agglomerates leaked out in the presence of salt during the reaction, leading to deactivation of the control channel.^46^,^47^ In contrast, Au NPs immobilised with portals of the CB[7] in the catalytic microreactor, did not leak out from the microchannel during the reaction,^33^,^34^ which was capable of maintaining the same yield for more than 300 h at a flow rate of 200 μL h^–1^.

The catalytic reduction of 4-nitrophenol by NaBH_4_ in the presence of Au NPs leading to 4-aminophenol, an important reactive intermediate for the photographic and pharmaceutical industries,^48^ led to similar results as the previous reaction (Fig. 3b and ESI Fig. S14†). Oxidation of the aromatic amine TMB by hydrogen peroxide in the presence of Au NPs, which is a highly sensitive method for the detection of H_2_O_2_ in biological fluids, was also tested.^49^Fig. 3c and ESI Fig. S15† show that Au NP microreactors efficiently catalysed the oxidation of TMB, giving 99% yield in 5 min at a flow rate of 200 μL h^–1^. A solution color change from colourless in the inlet tube to blue in the outlet tube was clearly observed. In contrast, both the control channel and bench reactions showed 0% conversion. The turnover frequency (TOF) values of the three reactions described above in the Au NP microreactors were calculated to be 171 ± 2, 178 ± 4 and 256 ± 6 s^–1^, respectively, which are 10^5^ times greater than the TOF for reactions on the bench (ESI Table S1†); moreover, these TOF values are much higher than those of most single-site Au NP heterogeneous catalysts,^35^,^36^,^39^,^41^ demonstrating excellent catalytic activity. Additionally, no detectable loss in the catalytic activity (TOF) was observed upon continuous operation for 300 h.

Three typical Pd NP catalysed cross-coupling reactions were examined for the CB[7]-based Pd NP microreactor, as depicted in Fig. 3d–f, ESI Table S1 and Fig. S16–21.† Interestingly, only the reactions in the microreactor led to pure products with high yields (99% for both Suzuki reactions and 85% for the Heck reaction), while reactions in both the blank microchannel and control channel did not yield any products. The bench reaction showed comparably low yields (40–50% for Suzuki reactions and 10% for the Heck reaction), all with various by-products. The high catalytic activities of the microreactors are attributed to the significant surface area to volume ratios of the NPs and the microchannel. The higher purity observed for the products may be due to the small channel diameter, which contributes to enhanced heat transfer, allowing high selectivity for desired products.^9^,^12^,^14^ The Pd microreactor gives rise to higher TOF than most single-site heterogeneous Pd catalysts (10–10^5^ times).^37^,^38^,^40^,^42^ TOF for both the Suzuki reactions in the microreactors were (1.3 ± 0.1) × 10^4^ s^–1^, and the TOF for the Heck reaction was 719 ± 2 s^–1^, at a flow rate of 100 μL h^–1^. Similarly, no loss in catalytic activity was observed upon continuous operation for 300 h.

## Conclusions

By taking advantage of the strong binding affinity of the CB[7] portal towards metallic NPs, as well as the ability of CB[7] to encapsulate small guest molecules such as viologen derivatives in its cavity, we have successfully fabricated CB[7]-based metallic NP catalytic microreactors. The metallic NPs were immobilised as a monolayer inside microchannels *via* supramolecular inclusion complexes (MV–silane@CB[7]) and subsequently attached to the carbonylated portal of the sequestered CB[7]. These microreactors exhibit several advantages. For instance, no further separation or recycling of the catalysts is required. More importantly, the substantially high surface area to volume ratios of both the microchannel and metallic NPs contribute to remarkable activity of the catalytic microreactors. All catalytic reactions investigated with the Au NP and Pd NP microreactors exhibited high yields with high TOF (up to 10^4^ s^–1^), significantly higher than those of most single-site heterogeneous NP catalysts^35^–^42^ and are above the values of relevant industrial applications (TOF values in the range of 10^–2^ to 10^2^ s^–1^).^50^ This CB[7]-based attachment methodology provides a powerful multifunctional platform for high performance flow chemistry, holding great promise in a variety of catalytic reactions.

## Conflicts of interest

There are no conflicts to declare.

## Supplementary Material

Supplementary information
