# Supplementary material for: Cucurbit[7]uril-based high-performance catalytic microreactors
Source: Nanoscale. 2018 Jul 27;10(31):14835–9. doi: 10.1039/c8nr02900h (PMC6088369; doi:10.1039/c8nr02900h)
Supplement: Supplementary file 1 [file NR-010-C8NR02900H-s001.pdf]

Electronic Supplementary Material (ESI) for Nanoscale.
This journal is © The Royal Society of Chemistry 2018

# Cucurbit[7]uril-based High-performance Catalytic Microreactors

**Xiaohe Ren[a], Ziyi Yu[b], Yuchao Wu[a], Ji Liu[a], Chris Abell[b], and Oren A. Scherman[a]***

[a] *Melville Laboratory for Polymer Synthesis, Department of Chemistry, University of Cambridge, Cambridge, CB2 1EW, United Kingdom. Fax: +44 1223 334866; Tel: +44 1223 331508; E-mail: oas23@cam.ac.uk.*

[b] *Department of Chemistry, University of Cambridge, Lensfield Road, Cambridge, CB2 1EW, UK. Fax: +44(0)1223 336455; E-mail: ca26@cam.ac.uk.*

## Contents

**S.1 Experimental** **S2**

S.1.1 Materials and general methods . . . . S2
S.1.2 Preparation of metallic catalyst nanoparticles . . . . S2
S.1.3 Synthesis and characterization of MV-silane . . . . S3
S.1.4 Preparation of PDMS microfluidic devices . . . . S3
S.1.5 Preparation of CB[7]-based catalytic microreactors . . . . S4

**S.2 Characterisation** **S5**

S.2.1 MV-silane and CB[7] complexation study . . . . S5
S.2.2 Metallic NPs TEM images . . . . S6
S.2.3 SEM characterisation of microreactors . . . . S6
S.2.4 AFM characterisation of Pd microreactors . . . . S7
S.2.5 Control channels AFM images . . . . S8
S.2.6 Au NPs microreactor using different loading concentrations . . . . S10

**S.3 Catalytic Activity Investigation** **S11**

S.3.1 Au NP catalytic microreactors . . . . S11
S.3.2 Pd NP catalytic microreactors . . . . S14
S.3.3 Turnover frequency calculation . . . . S19

## S.1 Experimental

### S.1.1 Materials and general methods

All the starting materials were purchased from Sigma Aldrich and used as received unless stated otherwise. CB[7] was prepared according to literature protocols.[1]

$^1$H NMR (400 MHz) spectrum was recorded using a Bruker Advance QNP 400. The binding constant of MV-silane and CB[7] was measured using Microcal$^{TM}$ iTC$_{200}$ (isothermal titration calorimetry). TEM images were obtained by a FEI Philips Tecnai 20 TEM under an accelerating voltage of 100 kV. Samples were prepared by applying one drop of the metallic NP solutions onto a Holey R carbon coated copper TEM grid (400 mesh), followed by drying overnight at room temperature. SEM images were obtained by a TESCAN MIRA3 FEG-SEM using a beam voltage of 5 kV under in-beam SE detector. Samples were prepared by cutting the microchannels from the middle, sticking onto SEM stubs and coating with 5 nm Chronium under vacuum using Quorum Technologies Q150T ES turbo-pumped sputter coater. AFM images were recorded using an Agilent Technologies 5500 AFM under AAC mode (acoustic intermittent contact mode). The AFM probes were purchased from Bruker Nano Inc. (MSNL, back layer of the cantilevers coated by 45 $\pm$ 5 nm Au). F triangle probes, with a spring constant of 0.6 N/m, resonant frequency of 125 kHz, tip radius of 20 nm by average and 60 nm at the maximum, were used. The AFM samples were prepared by carefully cutting the channels through the middle, then cutting it into square pieces with a thickness of around 1 mm. UV-*vis* spectra were obtained on a Varian Cary 4000 UV-*vis* spectrometer. High Pressure Liquid Chromatography (HPLC) was performed on a Varian 940-LC system. The Agilent Eclipse Plus C18 5 $\mu$m 4.6 x 150 mm column was used to analyse the resultant products. The gradient applied was from 95 : 5 water (0.1% trifluoroacetic acid): acetonitrile to 100% acetonitrile.

### S.1.2 Preparation of metallic catalyst nanoparticles

The metallic catalyst NPs were synthesized by rapid reduction of corresponding high valence metallic acids/salts in the presence of sodium borohydride. The Au NPs were synthesized by firstly mixing 1 *wt%* sodium citrate (2 mL) with 1 *wt%* chloroauric acid (1 mL) in 100 mL water, stirring and cooling in an ice bath for 10 min. Then 0.1 M sodium borohydride (10 mL, pre-cooled) was added rapidly, stirring for 1 h. Similarly, the Pd NPs were synthesized by the rapid addition of sodium borohyride (0.1 M, 4 mL, pre-cooled) to the cooled mixtures of 1 *wt%* sodium citrate (2 mL) with 10 mM sodium tetrachloropalladate (1 mL) in 100 mL water.

### S.1.3 Synthesis and characterization of MV-silane

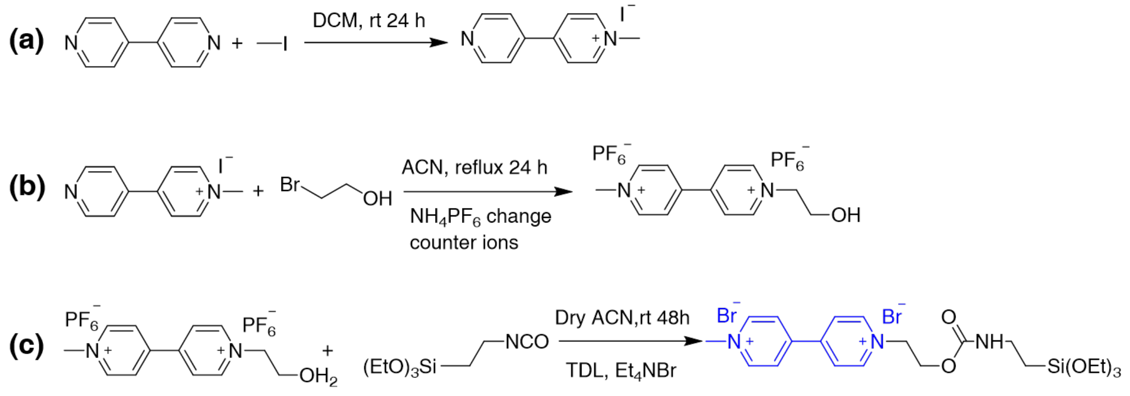


**Figure S1:** Reaction scheme of the synthesis of 1-(4,4-diethoxy-8-oxo-3,9-dioxa-7-aza-4-silaundecan-11-yl)-1'-methyl-[4,4'-bipyridine]-1,1'-diium (MV-silane).

(a) 4, 4'-bipyridine (20 g, 128 mmol) was dissolved in 200 mL dichloromethane (DCM). Methyl iodide (10 mL, 162 mmol) was well dispersed in 100 mL DCM, and then added dropwise to the bipyridine solution, allowing reaction at room temperature (rt) for 24 h. The product 1-methyl-4-(4-pyridinyl)pyridinium iodide (**A**, $MV^+$) was separated and washed with copious DCM, and then air dried (34.3 g, yield 79 %).

(b) $MV^+$ (**A**) (6.0 g, 20 mmol) was dissolved and refluxed in 300 mL acetonitrile (ACN). 2-bromoethanol (12.5 g, 100 mmol) was firstly dispersed in 15 mL ACN, and then added in to the $MV^+$ solution, refluxing for 24 h. The resultant product was separated and washed with copious ACN, then dissolved in 15 mL water and changed counter ions with ammonium hexafluorophosphate (4.16 g, 25.5 mmol) to obtain $C_{13}H_{16}N_2O^{2+} \cdot 2\,PF_6^-$ (**B**).

(c) **B** (0.9 g, 1.8 mmol) and 3-(triethoxysilyl)propyl isocyanate (4.45 g, 18 mmol) were dissolved in 20 mL dry ACN, with the addition of catalytic amount of dibutyltin dilaurate (TDL), stirring for 48 h under nitrogen. After that, tetraethylammonium bromide (2.3g, 10.9 mmol, dissolved in 3 mL dry ACN) was added in the resultant solution, stirring for 2 h. Then the product was separated and washed with ACN and copious diethyl ether (**C**, 1.36 g, yield 60%). $^1$H NMR (400 MHz, DMSO), 9.17 (*d*, 4H, $J_{AB}$=0.52Hz), 8.65 (*d*, 4H, $J_{BA}$=0.52Hz), 7.03 (*t*,1H, $J_{AB}$= 2.01Hz), 5.02 (*t*, 2H, $J_{BC}$=0.52 Hz), 4.50 (*t*, 2H, $J_{AC}$=1.26Hz), 4.45 (*s*, 3H), 3.78 (*q*, 6H), 2.89 (*t*, 2H, $J_{AB}$=1.68Hz), 1.21 (*t*, 9H, $J_{BC}$=0.31Hz), 0.9 (*t*, 2H, $J_{AC}$=0.99Hz). $^{13}$C NMR (400 MHz, DMSO), $\delta$ = 158.73, 150.87, 145.43, 126.95, 63.93, 59.98, 58.43, 48.83, 29.76, 20.45, 18.32 ppm. Mass Spectra, *m/z* = 449.3423 g/mol.

### S.1.4 Preparation of PDMS microfluidic devices

The microfluidic device was produced *via* standard soft lithography by pouring poly(dimethylsiloxane) (PDMS, 20 g) along with a crosslinker (Sylgard 184 elastomer kit, Dow Corning, pre-polymer : crosslinker = 10 : 1 by weight) onto a silicon wafer patterned with SU-8 photoresist.[2–4] It was then placed in vacuum for half an hour to remove dissolved gas. The PDMS was allowed to solidify at 90 °C for 12 h before being peeled off, after which inlets and outlets were generated using a biopsy punch. The enclosed microfluidic channels were formed by attaching the moulded PDMS replica onto a clean glass slide after exposure to oxygen plasma for 10 s in a Femto plasma cleaner.

The microchannels could be fabricated to various shapes and length (Figure S2 as examples), to satisfy the requirement of certain catalytic reactions, such as various inlets or gas protection. In this work, the PDMS microchannels with 40 $\mu$m diameter and 3 cm length was utilized.

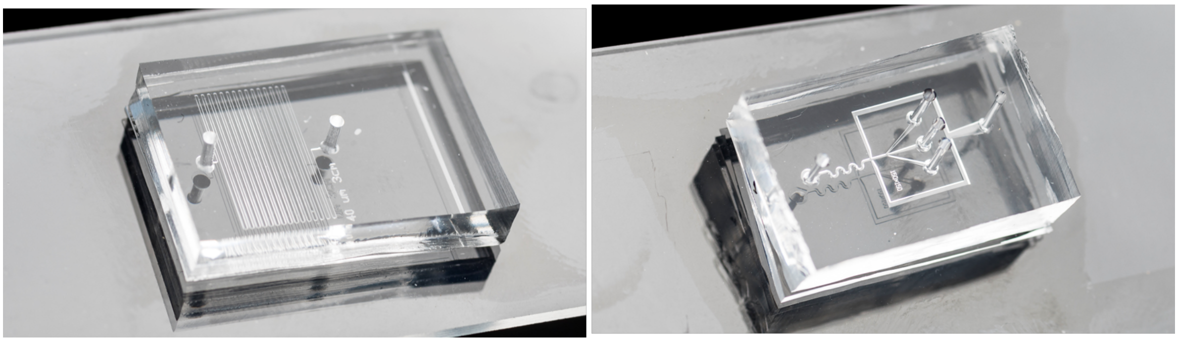

**Figure S2:** Pictures of example PDMS microchannels with various channel shapes and length.

### S.1.5 Preparation of CB[7]-based catalytic microreactors

The CB[7]-based Au NPs catalytic microreactors were prepared as follows:

(1) The blank PDMS microfluidic channels were activated by oxygen plasma for 10 s, after which the MV-silane@CB[7] solution (2.5 mM MV-silane, 1 mM CB[7], in 50 : 50 water : ethanol solvent) was injected and flowed through the microchannel at a flow rate of 300 $\mu$L h$^{-1}$ for 2 h, followed by washing with water for 1 h at the same rate.

(2) The prepared Au NPs solution was injected and flowed through the microchannel at a rate of 200 $\mu$L h$^{-1}$ for 1 h, followed by washing with water for 1 h at the same rate.

The CB[7]-based Pd NPs catalytic microreactors were prepared in the same approach, injecting the prepared Pd NPs solution in step (2) instead. Note that all the solutions were filtered using 200 nm filter tips before injecting into microchannels to avoid the blockage of the microchannels.

Control channels (**A** and **B**) were prepared by solely injecting MV-silane or CB[7] solutions in step (1). Control channel (**C**) was prepared by just conducting step (2), but without the injection of MV-silane@CB[7] inclusion complex solutions.

## S.2 Characterisation

### S.2.1 MV-silane and CB[7] complexation study

ITC was carried out to investigate the complexation of CB[7] and MV-silane (Figure S3), confirming strong binding between MV-silane and CB[7].

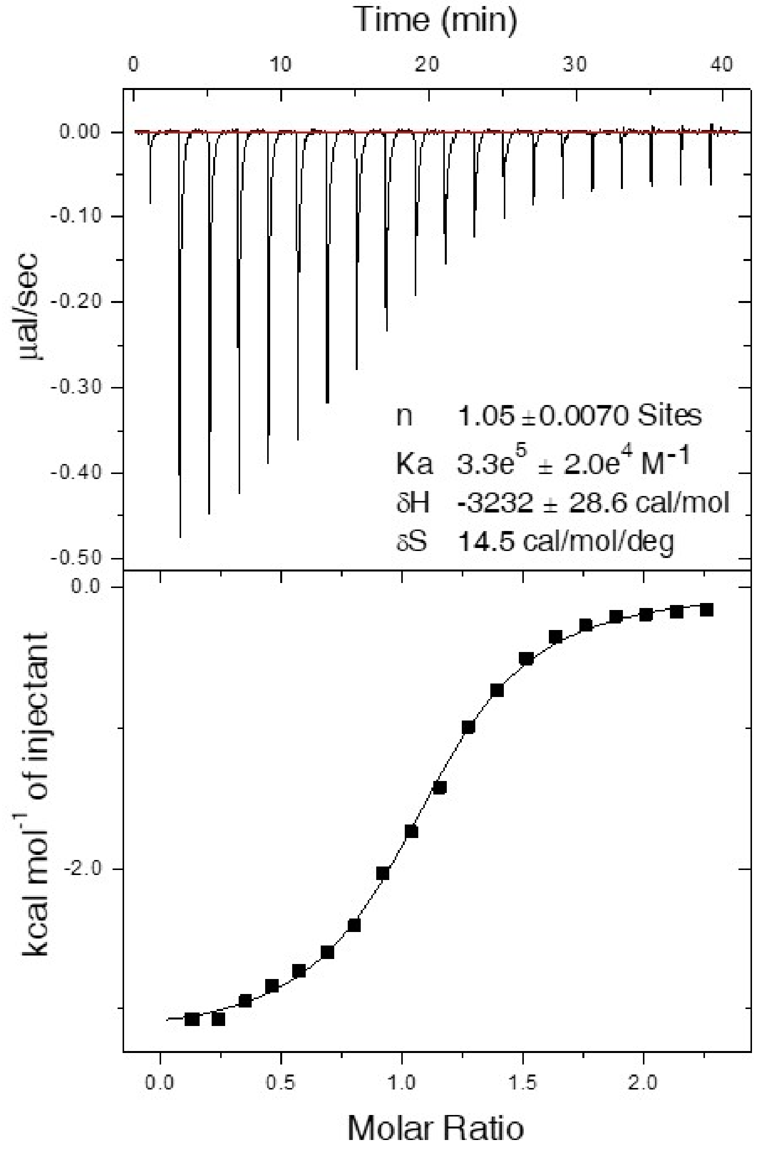


**Figure S3:** ITC results of the complexation between MV-silane and CB[7]. 1 mM MV-silane was injected into 0.05 mM CB[7] in buffer solution at 25 °C. 50 mM sodium acetate buffer, pH=4.6, was used.

### S.2.2 Metallic NPs TEM images

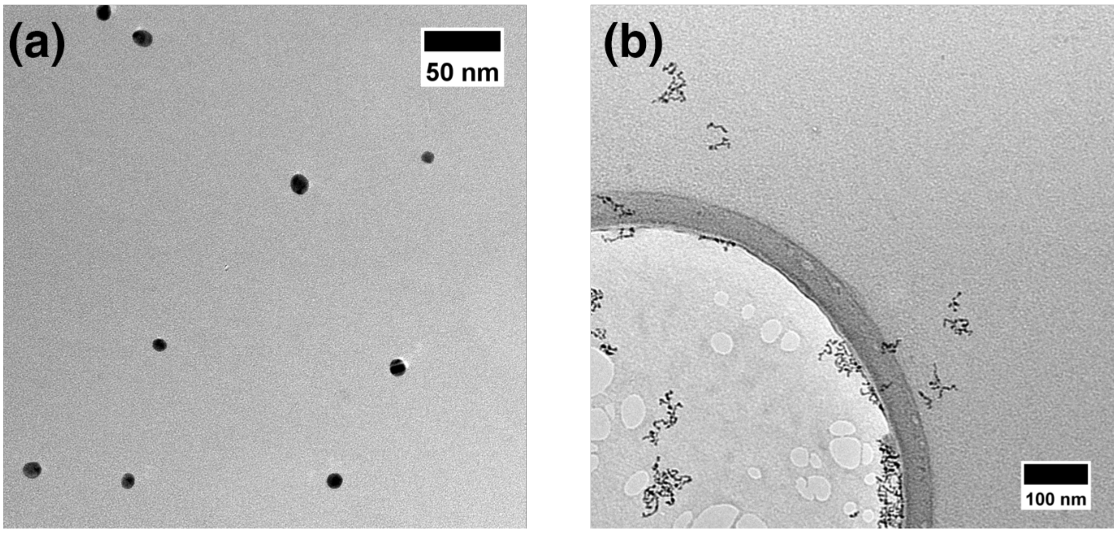


**Figure S4:** TEM images of (a) Au NPs (6.8 ± 2.1 nm) and (b) Pd NPs (3.7± 0.8 nm)

### S.2.3 SEM characterisation of microreactors

As seen in Figure S5 and S6, control (**A**) shows severe agglomeration of metallic particles on a scale of micrometers (Figure S5c and S6f). Control (**B**) and (**C**) resemble the blank channel, since all the NPs were flushed out of the microchannels (Figure S5d-e, S6d-e).

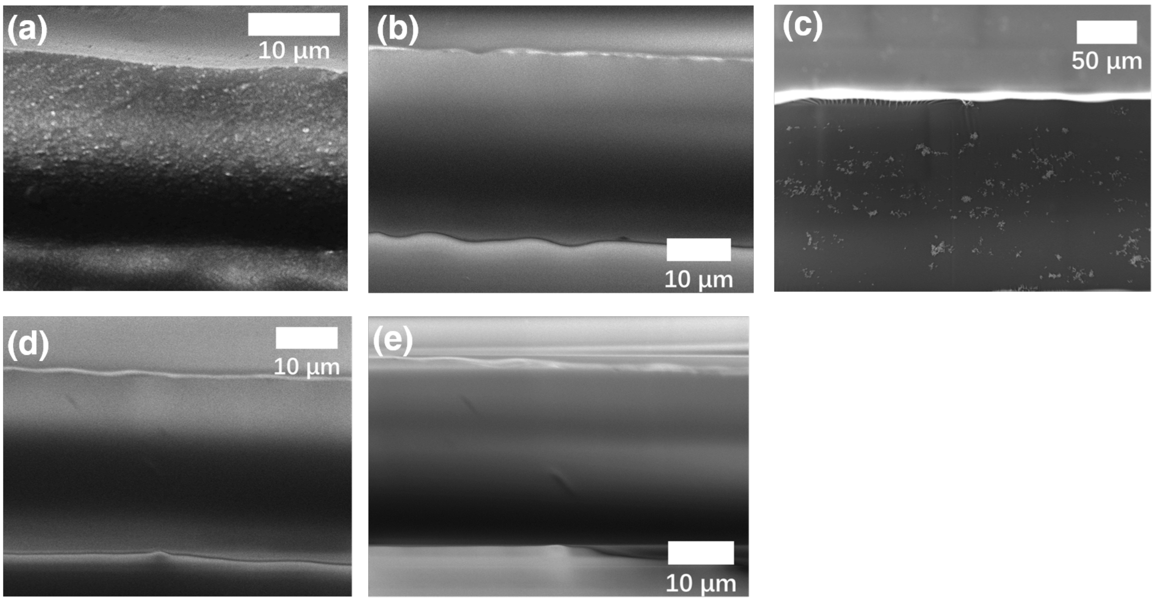


**Figure S5:** SEM images of (a) CB[7]-based Au NP catalytic microreactor, (b) blank PDMS microchannel, (c) control channel (**A**), (d) control channel (**B**), (e) control channel (**C**).

Note: Control channels (**A** and **B**) were prepared by solely injecting MV-silane or CB[7] solutions, followed by metallic NP solutions. Control channel (**C**) was prepared by just injecting metallic NPs.

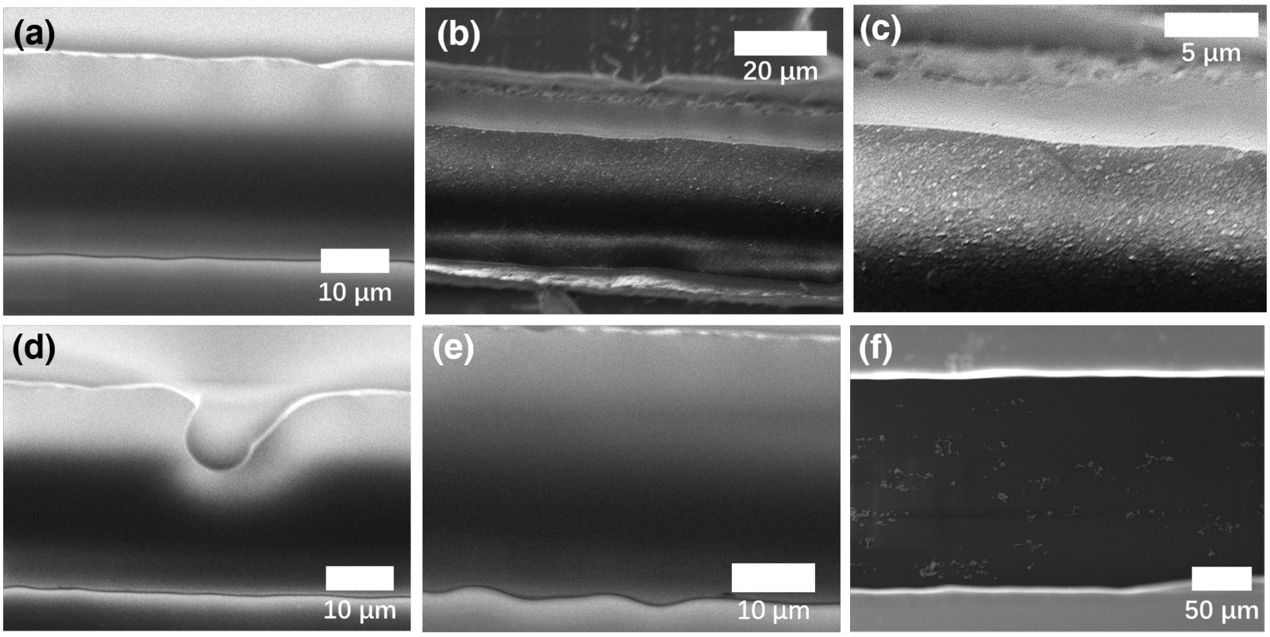


**Figure S6:** SEM images of (a) blank PDMS microchannel, (b) CB[7]-based Pd NP catalytic microreactor, (c) zoom in image of (b), (d) control channel (**A**), (e) control channel (**B**), (f) control channel (**C**), where agglomerates in micrometer scale (no catalytic activity) were observed.

### S.2.4 AFM characterisation of Pd microreactors

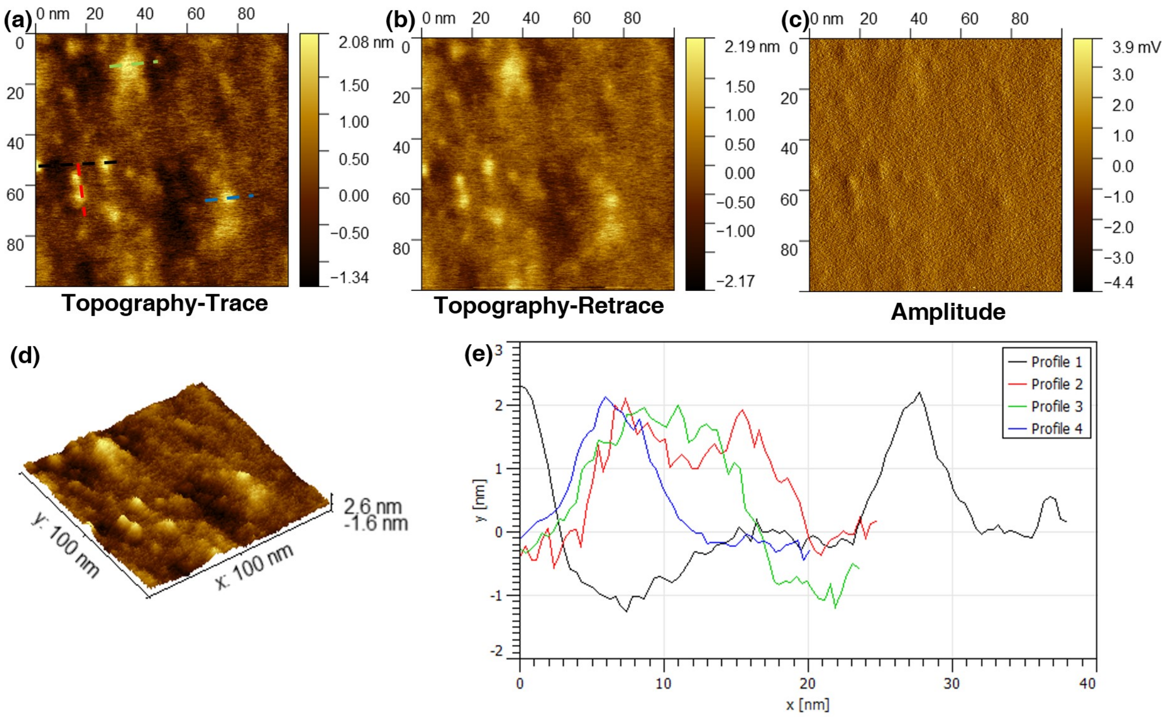


**Figure S7:** AFM images of Pd NP catalytic microreactor, where good immobilization of Pd NPs was observed. (a) AFM topography images (trace); (b) AFM topography image (retrace); (c) AFM amplitude image; (d) AFM 3D view image; (e) Profile of the dashed lines indicated in (a).

### S.2.5 Control channels AFM images

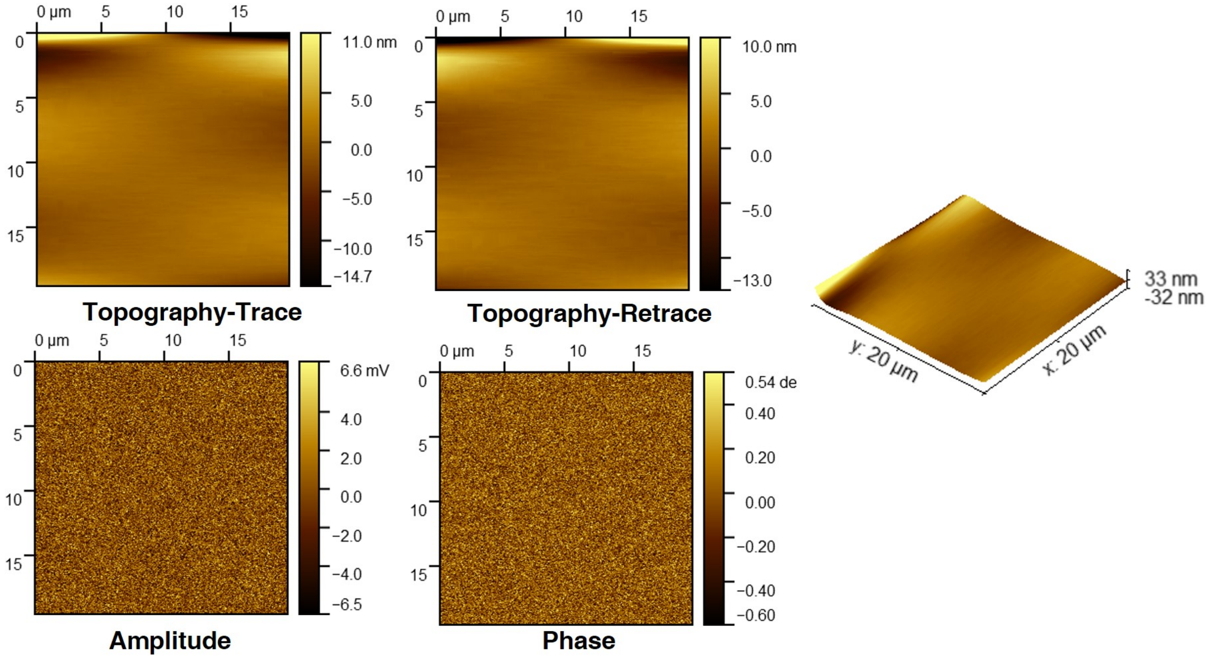


**Figure S8:** AFM images of blank PDMS microchannels (roughness = 0.06 nm).

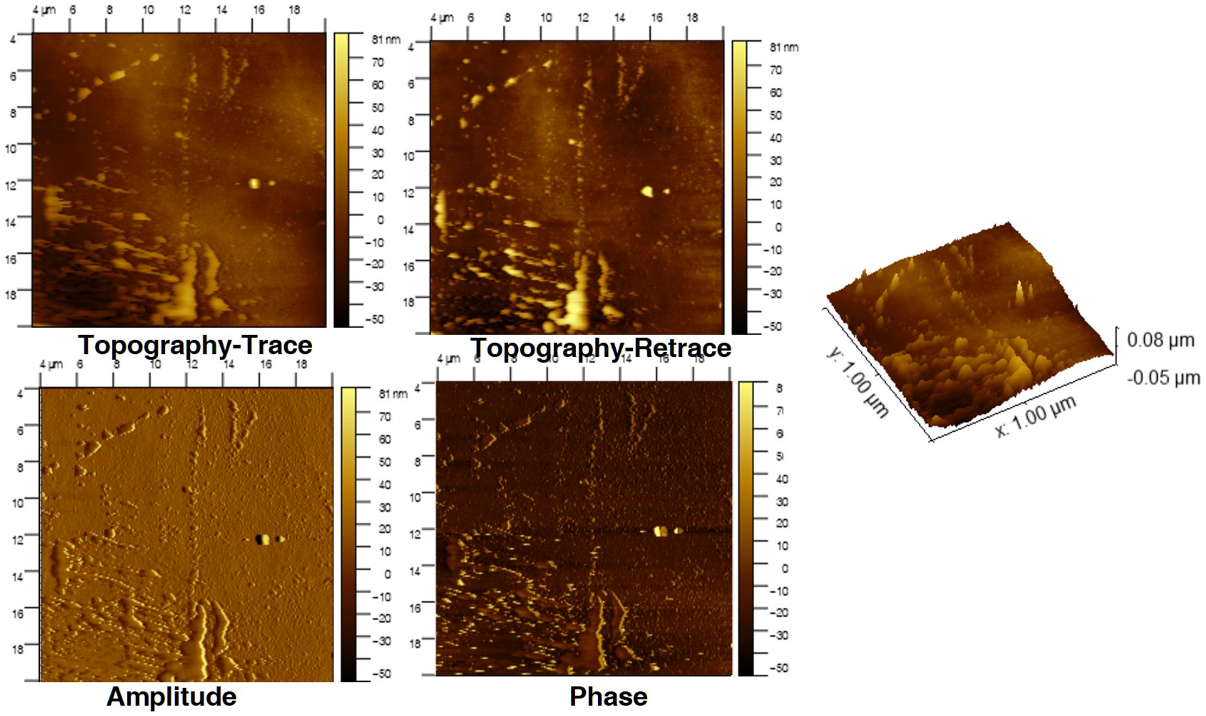


**Figure S9:** AFM images of Au NP control channel (**A**, just injecting MV-silane and then metallic NP solutions, but without CB[7] solutions), where serious agglomeration of Au particles was observed. Roughness = 2.1 $\mu$m.

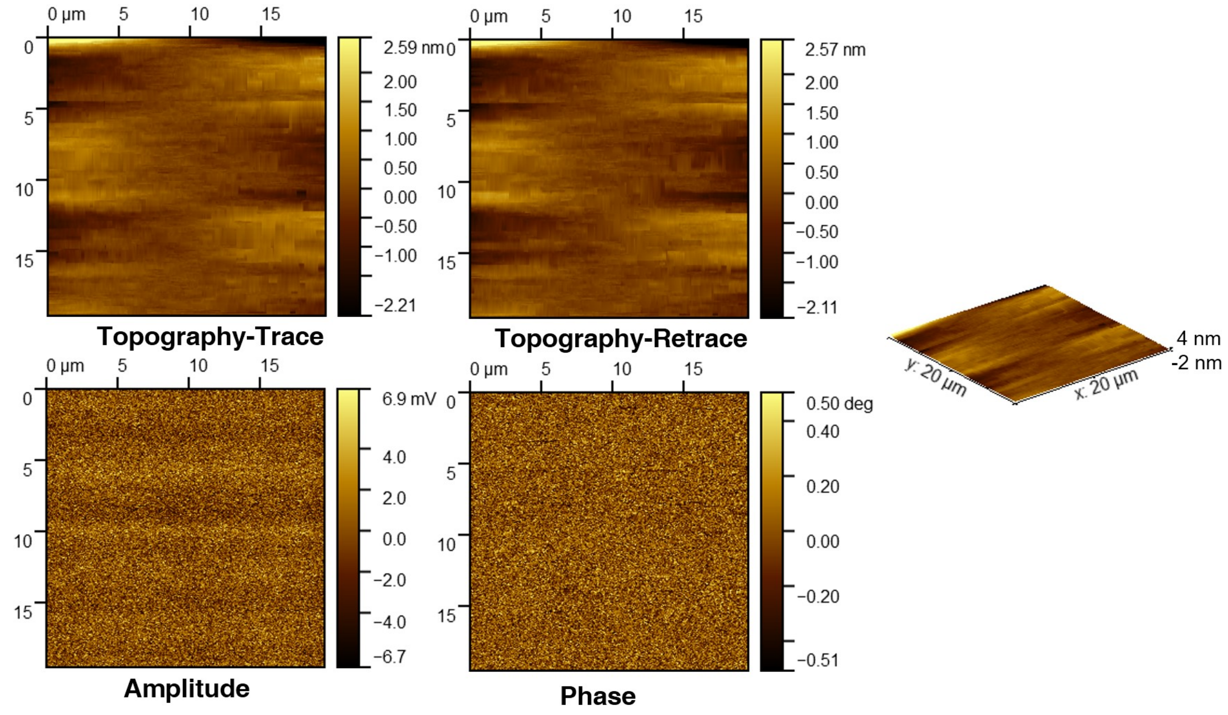


**Figure S10:** AFM images of Au NP control channel (**B**, prepared by just injecting CB[7] and then metallic NP solutions, but without the injection of MV-silane solutions), where no Au NPs were observed.

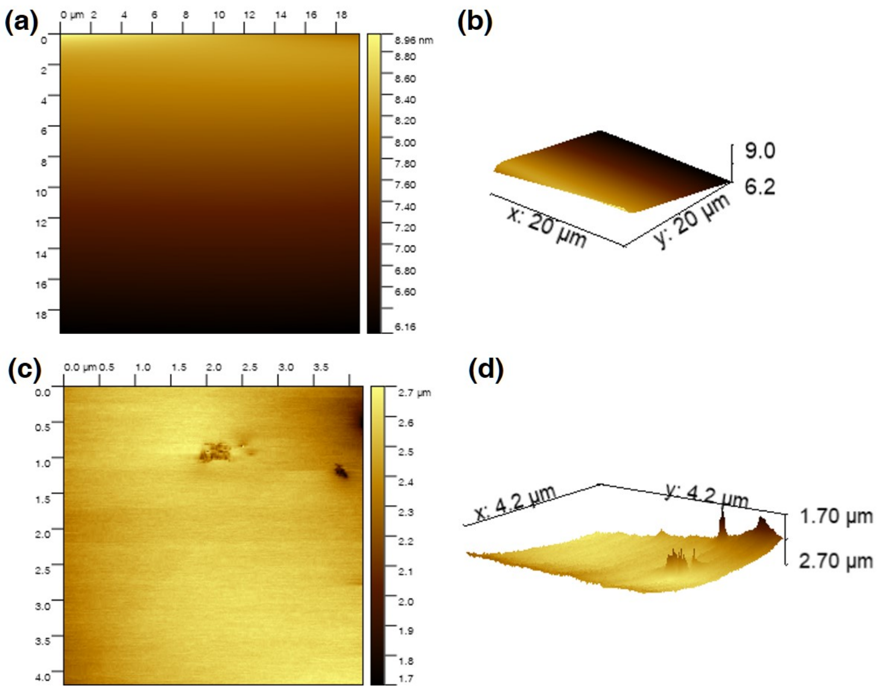


**Figure S11:** Topography and 3D view AFM images of Pd control channels. (a, b) were prepared by only injected Pd NPs, where no NP was observed in the microchannel. (c, d) were prepared by only injected MV-silane and Pd NP solutions, without CB[7] solutions. The agglomerates up to micrometer scale were observed, however, showing no catalytic activity.

### S.2.6 Au NPs microreactor using different loading concentrations

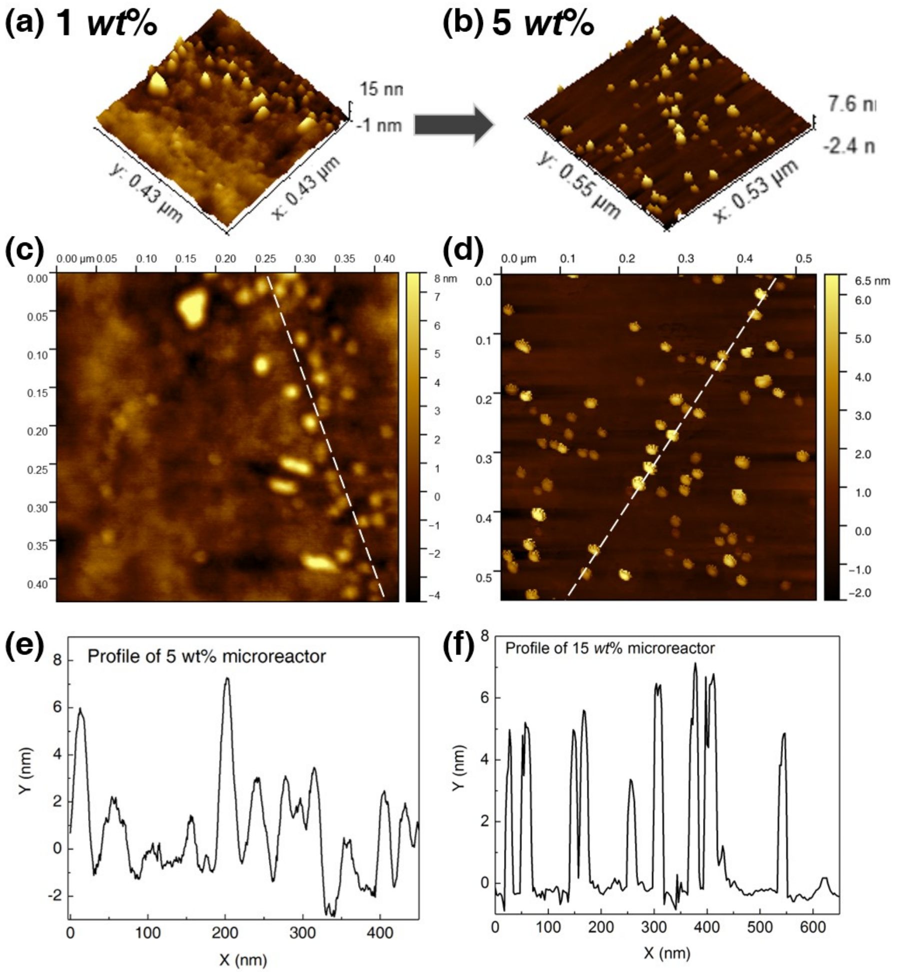


**Figure S12:** AFM 3D view images (a, b) and topography images (c, d) of Au NP catalytic microreactors prepared using 1 *wt%* and 5 *wt%* Au NP solutions, respectively. (e, f) show the profiles of the dashed lines indicated in (c, d) respectively. An increase of the loading NP density using increased concentration was observed.

## S.3 Catalytic Activity Investigation

**Table S1:** Summary of representative reactions catalysed by CB[7]-based Au NPs or Pd NPs catalytic microreactors.

| Entry | Reaction Mode | Yield (%) | TOF[a] ($s^{-1}$) |
|---|---|---|---|
| 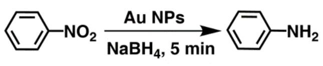 | microreactor | 99[b] | $(1.7 \pm 0.1) \times 10^{2}$ |
| | control channel[c] | 0[d] | 0 |
| | bench [e] | 50 | $(4.2 \pm 0.2) \times 10^{-3}$ |
| 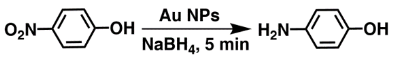 | microreactor | 99 | $(1.8 \pm 0.1) \times 10^{2}$ |
| | control channel | 0[f] | 0 |
| | bench | 60 | $(5.0 \pm 0.3) \times 10^{-3}$ |
| 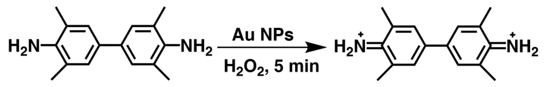 | microreactor | 99 | $(2.5 \pm 0.1) \times 10^{2}$ |
| | control channel | 0 | 0 |
| | bench | 0 | 0 |
| 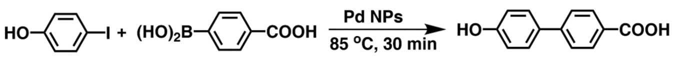 | microreactor | 99[g] | $(1.3 \pm 0.1) \times 10^{4}$ |
| | control channel | 0 | 0 |
| | bench | 40 | $(1.0 \pm 0.1) \times 10^{3}$ |
| 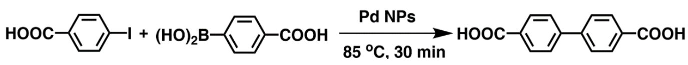 | microreactor | 99 | $(1.3 \pm 0.1) \times 10^{4}$ |
| | control channel | 0 | 0 |
| | bench | 50 | $(1.3 \pm 0.1) \times 10^{3}$ |
| 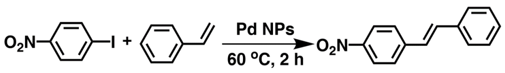 | microreactor | 85 | $(7.2 \pm 0.1) \times 10^{2}$ |
| | control channel | 0 | 0 |
| | bench | 10 | $(7.1 \pm 0.1) \times 10^{-4}$ |

[a] Turnover frequency (TOF) suggests remarkable catalytic activity of the microreactors, which is much above most relavant industrial reactions (TOF sit in between $10^{-2}$ to $10^{2}$ $s^{-1}$).[5] Moreover, the TOF of the microreactors remained unchanged for more than 300 h at a flow rate of 200 $\mu$L $h^{-1}$. [b] The yield of three Au NPs reactions were determined from UV-vis absorption analysis. [c] Control channels were prepared by only injecting and flowing MV-silane and metallic NP solutions through the microchannel sequentially, but without CB[7] solutions. [d] The first run gave rise to 50% yield; however, later reaction cycles produced 0% yield. [e] Bench reactions were carried out under approximately similar conditions, using free metallic NPs as the catalyst instead. [f] The first run gave rise to 60% yield; however, later reaction cycles quickly decreased to 0% yield. [g] The yield of three Pd NPs reactions were determined from HPLC analysis.

### S.3.1 Au NP catalytic microreactors

#### S. 3.2.1 Reduction of nitrobenzene by $NaBH_4$

6 mL deionised water, 0.5 mL 1 mM nitrobenzene and 2 mL 50 mM $NaBH_4$ were mixed, injected and flowed through the microreactor, control channel (**A**) and blank microchannel, all at a flow rate of 200 $\mu$L $h^{-1}$. The bench reaction was carried out at the same experimental condition, using Au NP (6.8 $\pm$ 2.1 nm, 1 *wt%* 100 $\mu$L ) as the catalyst instead. The resultant products were analysed using UV-*vis* spectroscopy. Yield was deduced from the reactant absorption peak (nitrobenzene, 270nm). The microreactor can be operated continuously for 300 h without detectable loss of the yield and TOF.

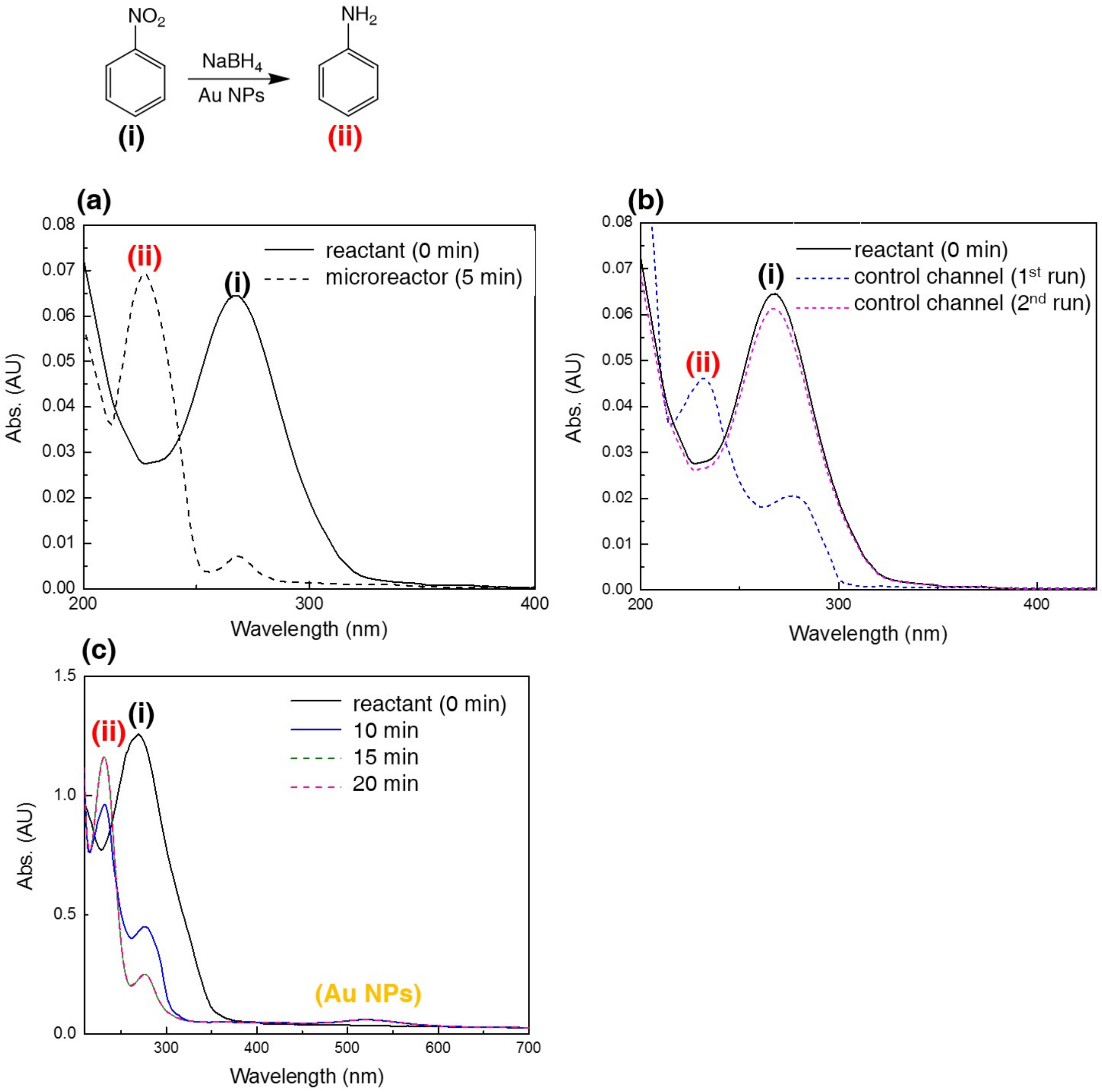


**Figure S13:** UV-*vis* spectra of (a) Au NP catalytic microreactor before and after reaction, yielding 99% after 5 min. (b) Au NP control channel: the first run gave 50% yield (the blue dashed line), then subsequent reaction cycles gave 0% yield (the pink dashed line) likely due to severe leakage of the gold agglomerated particles as a function of salts presented in the reaction. (c) The bench reaction with Au NPs led to a yield of 50% after 5 min and 99% after 15 min. No further increase of the product peak (ii) was observed after 15 min. The peak at 530 nm was caused by the remaining Au NPs in the reaction solution. The Au NPs used for the bench reaction were difficult to be separated and recycled for further use.

## S. 3.2.2 Reduction of nitrophenol by $NaBH_4$

Similar to previous reaction, the products were analysed using UV-*vis* spectroscopy.

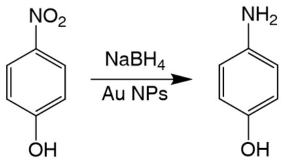


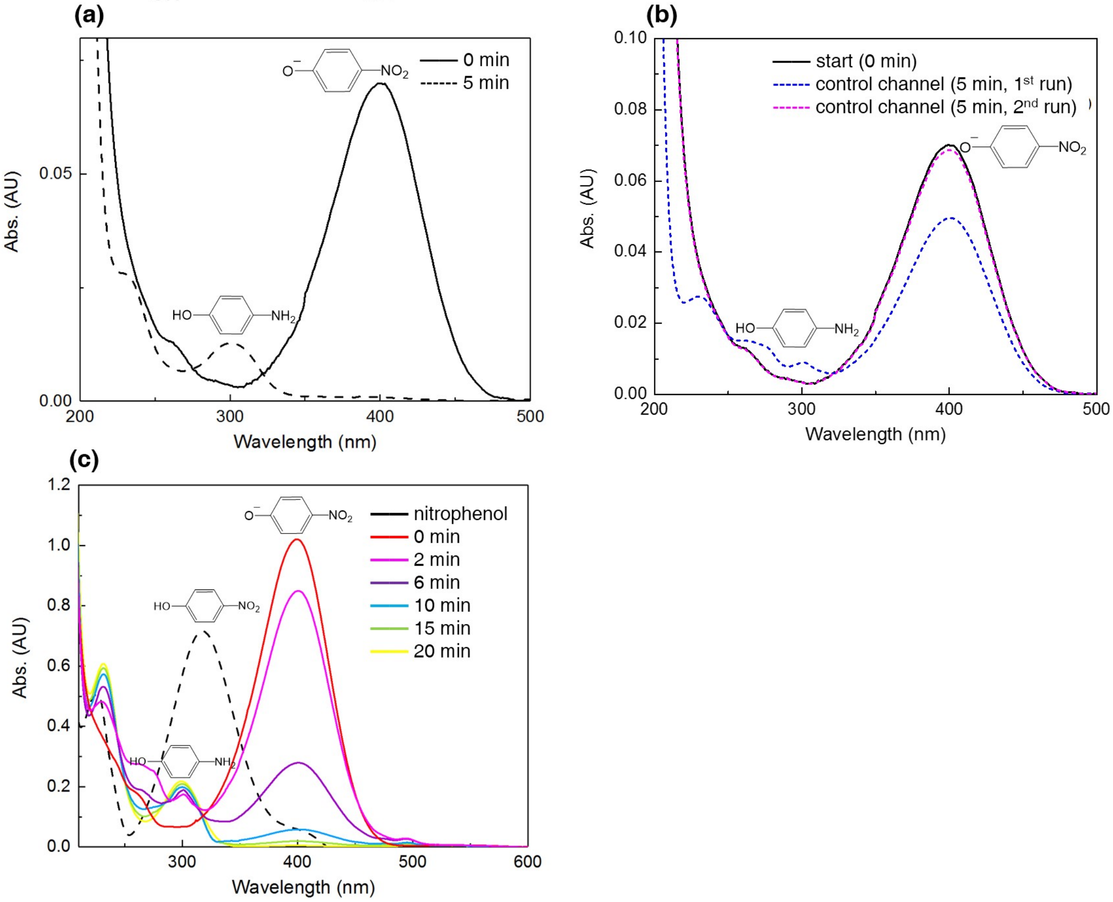


**Figure S14:** UV-*vis* spectra of (a) Au NP catalytic microreactor before and after reaction, yielding product in 99% after 5 min. (b) Au NP control channel: the first run gave 60% yield (the blue dashed line); however, the yield quickly decreased to 0% in subsequent reaction cycles (the pink dashed line). (c) The bench reaction with Au NPs led to a yield of 60% after 5 min and 99% till 20 min. The Au NPs used for the bench reaction were difficult to be separated and recycled for further use.

## S. 3.2.3 Oxidation of TMB by $H_2O_2$

The mixture of 3,3',5,5'-tetramethylbenzidine (TMB, 1 mM, in 3 mL ethanol/water 1 : 1 solvent) and $H_2O_2$ (4 M in 3 mL water) was injected and flowed through the microreactor, control channel (**A**) as well as blank microchannel, at a flow rate of 200 $\mu$L $h^{-1}$. A solution color change from colourless in the inlet tube to blue in the outlet tube was clearly observed in the catalytic microreactor. The resultant products were analysed using UV-*vis* spectroscopy. Yield was deduced from the product absorption peak (oxidized TMB, 652 nm). The microreactor can be operated continuously for 300 h without detectable loss of the yield and TOF.

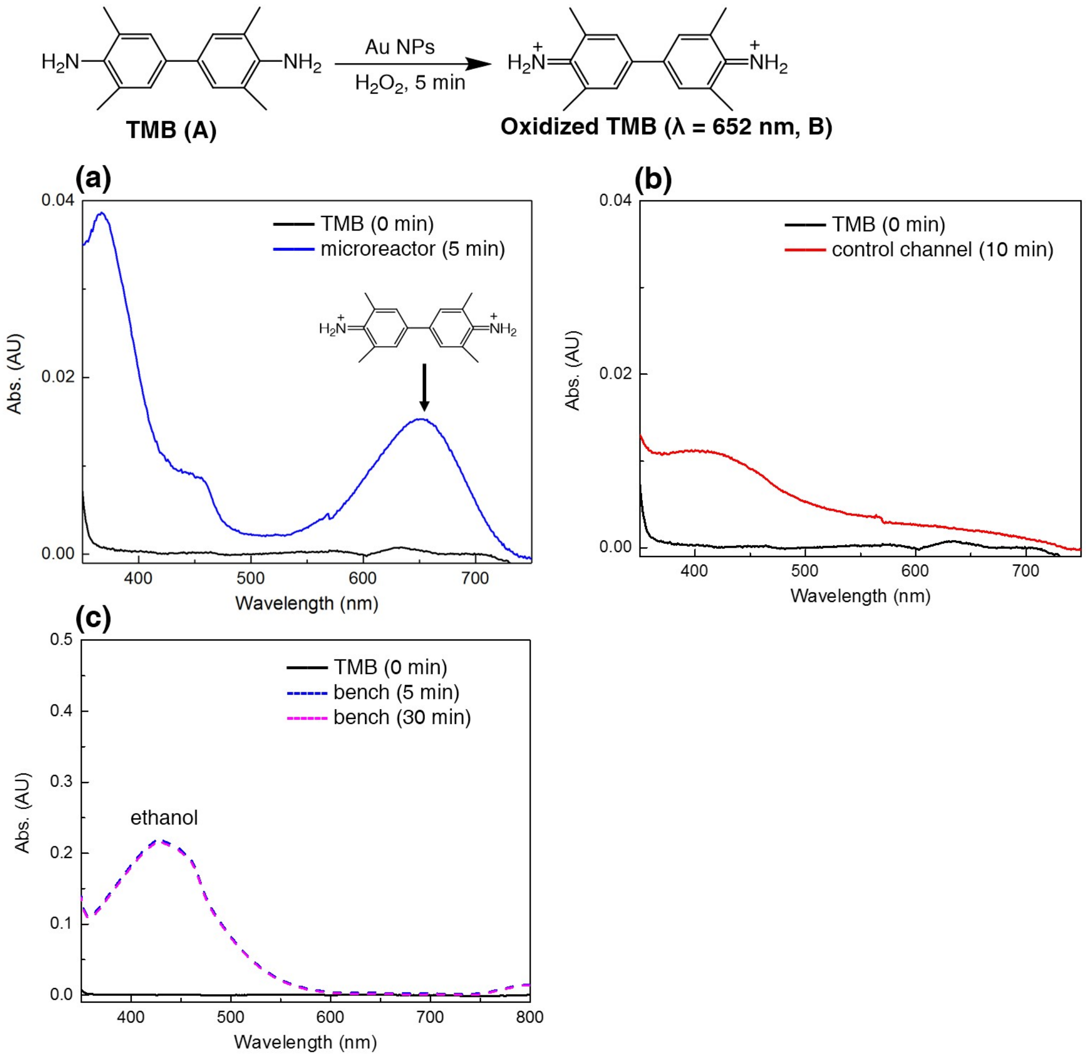


**Figure S15:** UV-*vis* spectra of (a) Au NP catalytic microreactor before and after reaction, yielding 99% after 5 min. (b) control channel did not produc any product after 10 min. (c) The bench reaction with Au NPs did not produce any product after 5 min and even 30 min. The wide peak around 400 nm came from the ethanol solvent.

## S.3.2 Pd NP catalytic microreactors

### S. 3.3.1 Suzuki reaction 1

4-iodophenol (100 mM) and potassium carbonate (200 mM) in 10 mL ethanol/water 1 : 1 solvent was preheated at 85 °C and mixed with 4-carboxyphenylboronic acid (150 mM in 10 mL ethanol/water 1 : 1 solvent). The reaction mixture was injected and flowed through the preheated microreactor and control channel (**A**) at a flow rate of 100 $\mu$L h$^{-1}$, heating and reacting at 85 °C for 30 min. The bench reaction was carried out using Pd NP (3.7 ± 0.8 nm, 1 *wt%* 100 $\mu$L) to catalyse 2 mL reaction mixture at 85°C for 30 min. The resultant products were analysed using HPLC. The yield was deduced from HPLC calibration line of the reactant (4-iodophenol). The microreactor can be operated continuously for 300 h without detectable loss of the yield and TOF.

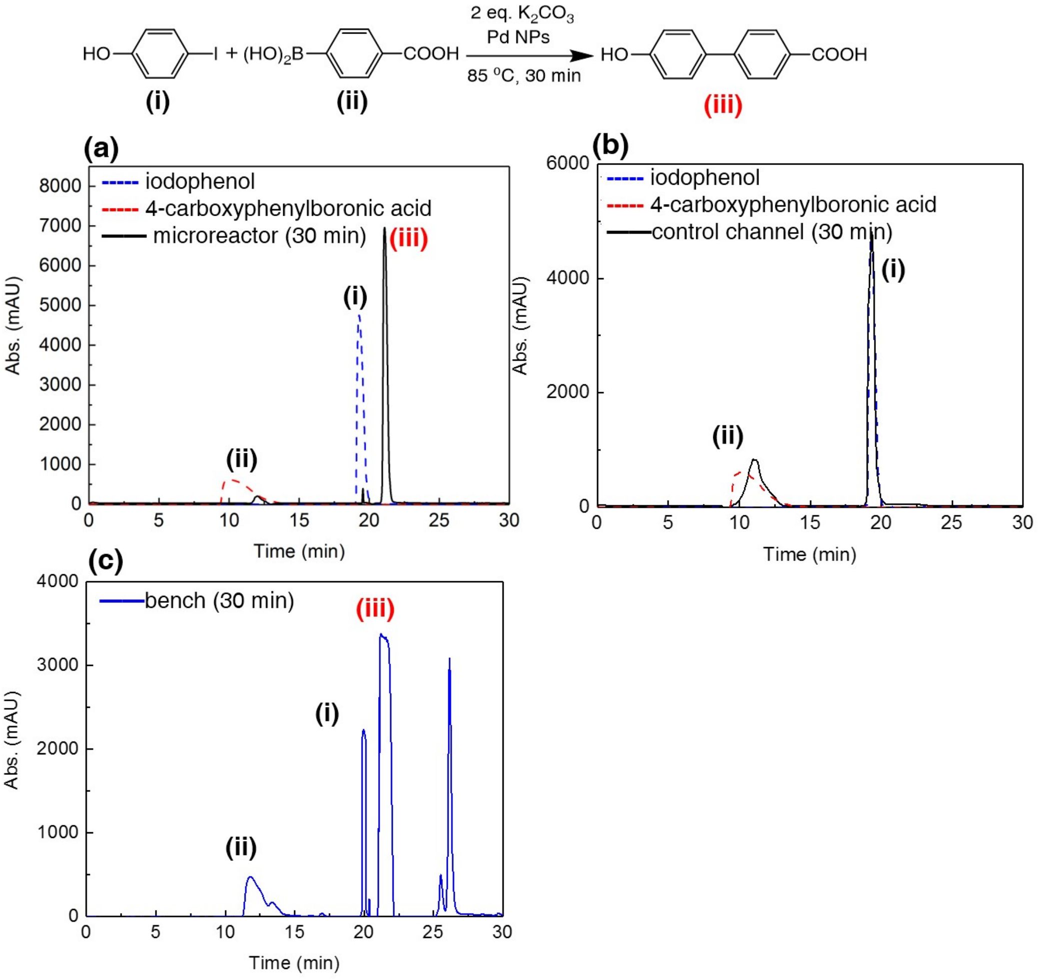


**Figure S16:** HPLC spectra of (a) reactants (dashed lines) and after reaction in Pd NP catalytic microreactor for 30 min, leading to 99% yield (solid line). (b) The Pd NP control channel did not yield any product after 30 min. Only the peaks of the reactants were observed. (c) The bench reaction with Pd NPs gave 40% yield after 30 min, however, with various byproducts (peaks between 25 to 30 min). The Pd NPs used in the bench reaction were difficult to be separated and recycled for further use.

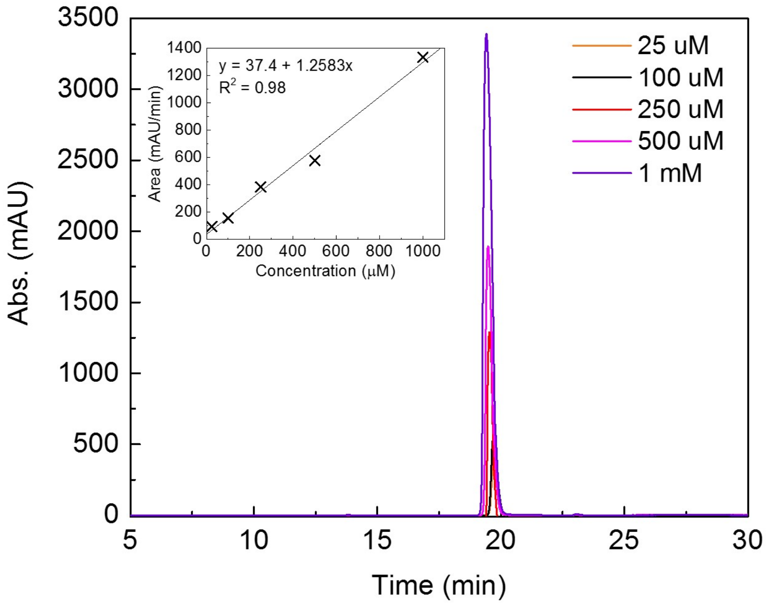


**Figure S17:** HPLC calibration line of 4-iodophenol.

**S. 3.3.2 Suzuki reaction 2**

Similar to previous reaction, the products were analysed using HPLC. The yield was deduced from HPLC calibration line of the reactant (4-iodobenzoic acid).

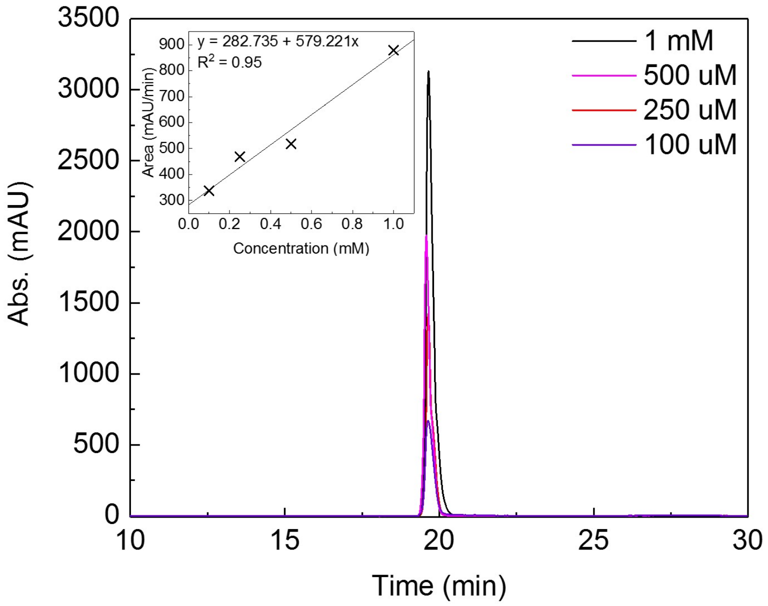


**Figure S18:** HPLC calibration line of 4-iodobenzoic acid.

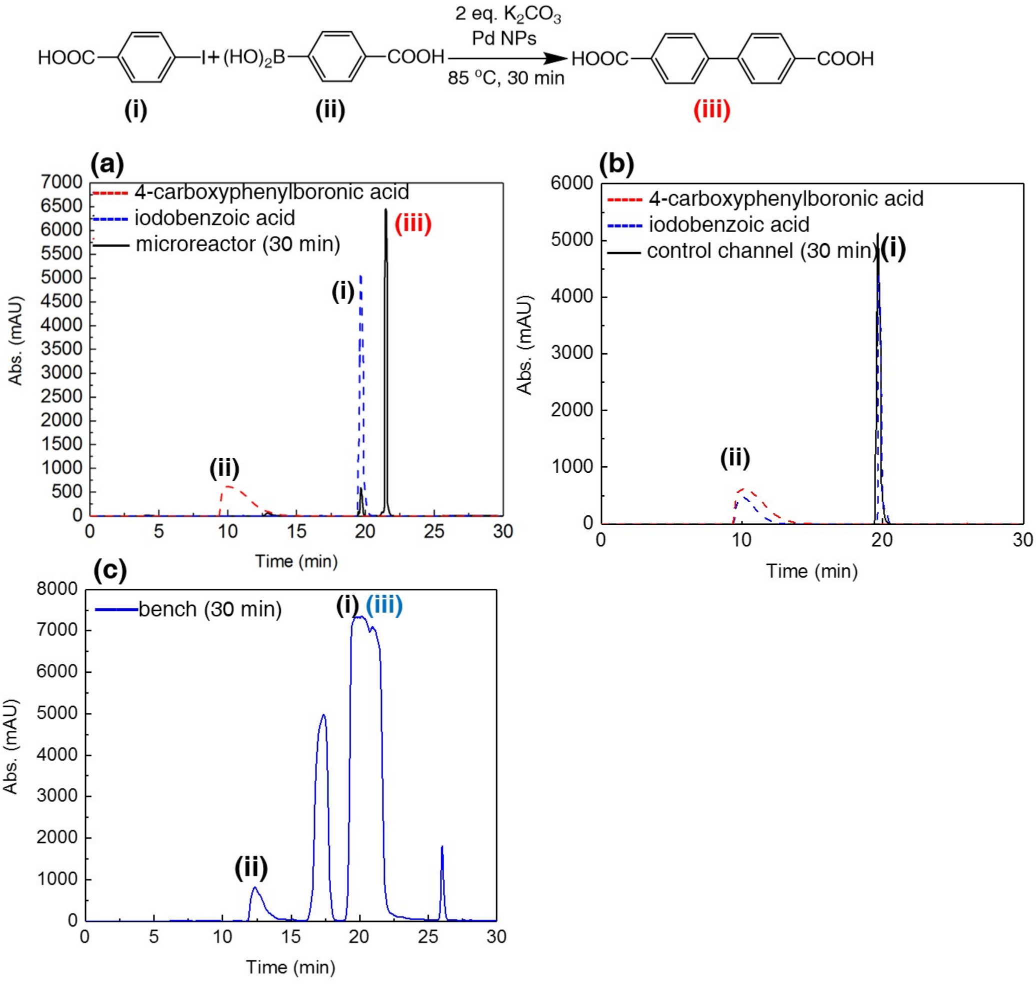


**Figure S19:** HPLC spectra of (a) reactants (dashed lines) and after reaction in Pd NP catalytic microreactor for 30 min, leading to 99% yield (solid line). (b) The Pd NP control channel did not produce any product after 30 min. Only the peaks of the reactants were observed. (c) The bench reaction with Pd NPs gave 50% yield after 30 min, however, with various byproducts (peaks at 17 and 27 min). The Pd NPs used in the bench reaction were difficult to be separated and recycled for further use.

### S. 3.3.3 Heck reaction

1-iodo-4-nitrobenzene (13.3 mM), styrene (16.6 mM), potassium carbonate (26.6 mM) and tetrabutylammonium bromide (0.8 mM) were dissolved in 7.5 mL water/DMF 1 : 2 solvent and preheated at 60 °C. The reaction mixture was injected and flowed through the preheated microreactor and control channel (**A**) at a flow rate of 100 $\mu$L h$^{-1}$, heating and reacting at 60 °C for 2 h. The bench reaction was carried out using Pd NP (3.7 ± 0.8 nm, 1 *wt%* 100 $\mu$L) to catalyse 2 mL reaction mixture at 60°C for 4 h. The resultant products were analysed using HPLC. The yield was deduced from HPLC calibration line of the reactant (1-iodo-4-nitrobenzene). The microreactor can be operated continuously for 300 h without detectable loss of the yield and TOF.

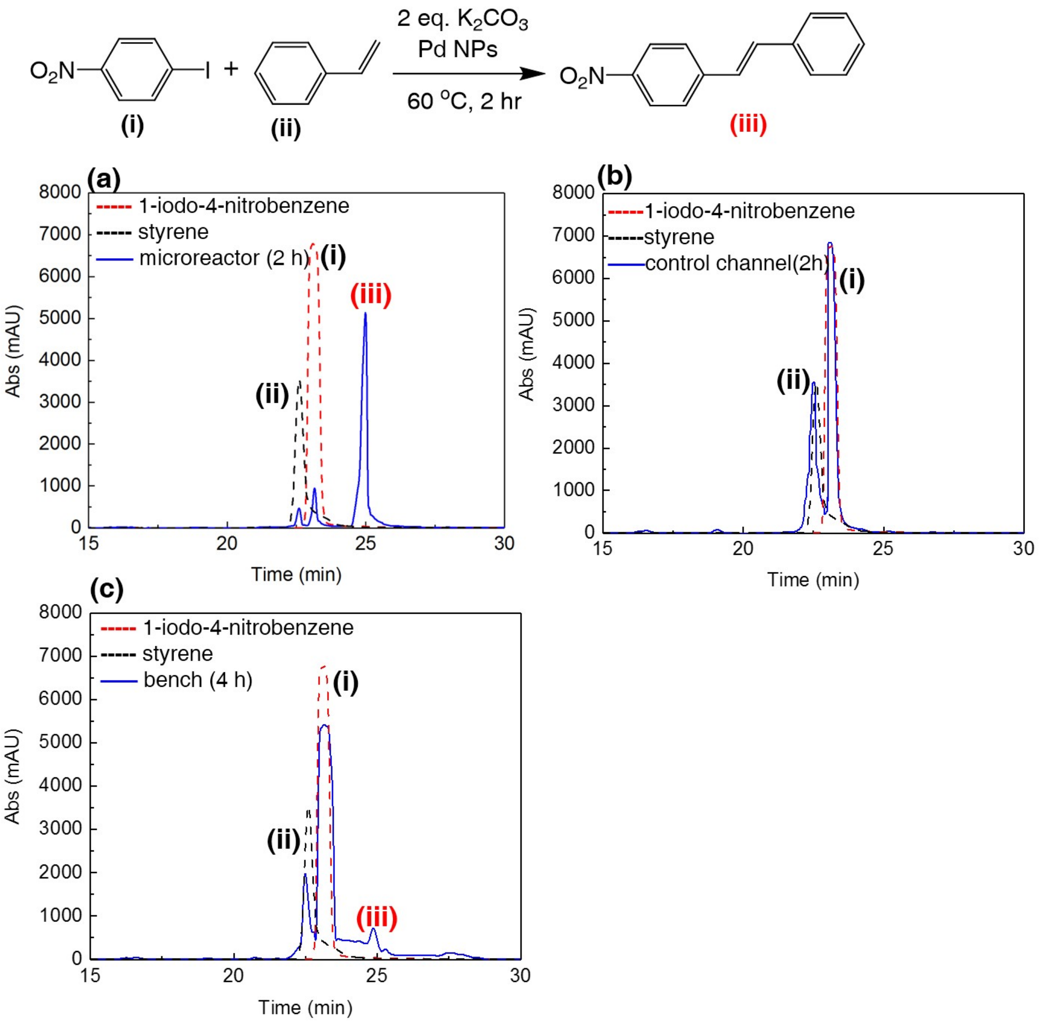


**Figure S20:** HPLC spectra of (a) reactants (dashed lines) and after reaction in Pd NP catalytic microreactor for 2 h, leading to 85% yield (solid line). (b) The Pd NP control channel did not produce any product after 2 h. The peaks remained the same with the reactants. (c) The bench reaction with Pd NPs gave 10% yield after 4 h.

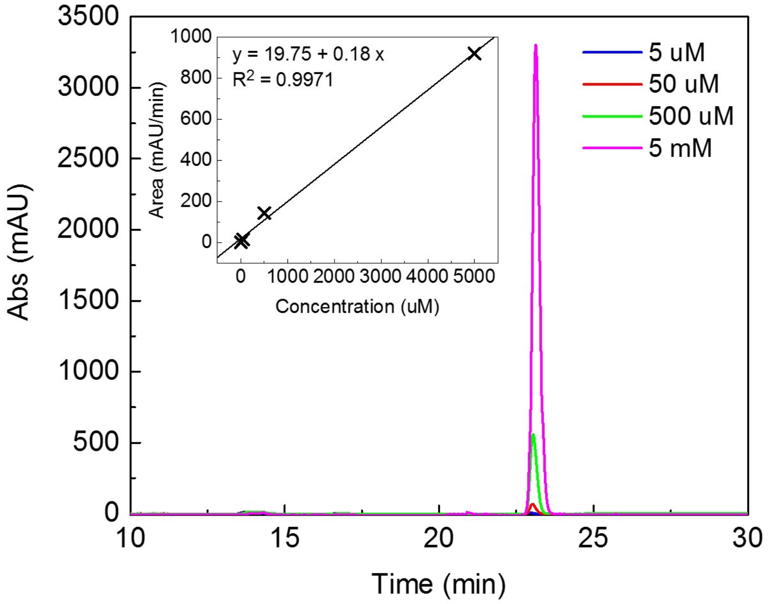


**Figure S21:** HPLC calibration line of 1-iodo-4-nitrobenzene.

### S.3.3 Turnover frequency calculation

Turnover frequency (TOF) is the total number of moles transformed into the desired product by one mole of active site per second. The larger the TOF, the more active the catalyst.[6]

$$TOF = \frac{Moles\ of\ product}{Moles\ of\ catalyst \quad \times \quad Reaction\ time}$$

The TOF for Au NP catalytic microreactor catalysing the reduction of nitrobenzene was calculated as follows:

$$\text{Concentration of reactant} = \frac{1mM \times 0.5mL}{8.5mL} = 58.8\ \mu M$$

$$\text{Moles of reactant} = 58.8\mu M \times 200\mu L/h \times 5min = 3.56 \times 10^{-10}\ \text{mol}$$

$$\text{Moles of product} = (99\% \pm 1\%)\ \text{x moles of reactant} = (3.52 \pm 0.04)\ \text{x}\ 10^{-10}\ \text{mol}$$

Number of catalyst in the microreactor with a diameter of 40 $\mu$m diameter and length of 3 cm = $\pi$d x 3 cm x density of the immobilised NPs = 4.14 x $10^9$

$$\text{Mole of catalyst} = 4.14\ \text{x}\ 10^9 \div N_A = 6.89\ \text{x}\ 10^{-15}$$

So

$$\text{TOF} = \frac{(3.52 \pm 0.04) \times 10^{-10} mol}{6.89 \times 10^{-15} \times 300s} = 171 \pm 2$$

TOF for other reactions were calculated by the same method.

## References


[1] J. Kim, I. S. Jung, S. Y. Kim, E. Lee, J. K. Kang, S. Sakamoto, K. Yamaguchi and K. Kim, *J. Am. Chem. Soc.*, 2000, **122**, 540–541.

[2] D. Qin, Y. Xia and G. M. Whitesides, *Adv. Mater.*, 1996, **8**, 917–919.

[3] D. C. Duffy, J. C. McDonald, O. J. Schueller and G. M. Whitesides, *Anal. Chem.*, 1998, **70**, 4974–4984.

[4] Z. Yu, J. Zhang, R. J. Coulston, R. M. Parker, F. Biedermann, X. Liu, O. A. Scherman and C. Abell, *Chem. Sci.*, 2015, **6**, 4929–4933.

[5] H. Jens, in *Industrial Catalysis: A Practical Approach, 2nd ed.*, Wiley-VCH: weinheim, 2006, ch. Homogeneously catalyzed industrial processes, pp. 59–82.

[6] S. Kozuch and J. M. Martin, *ACS Catal.*, 2012, **2**, 2787–2794.
